# The role and mechanisms of the vascular microenvironment in metabolic dysfunction– associated fatty liver disease

**DOI:** 10.3389/fendo.2026.1916728

**Published:** 2026-10-30

**Authors:** Rui Li, Xiaojuan Huang, Shengbiao Li, Tongtong Zhang

**Affiliations:** 1 Obesity and Metabolism Medicine-Engineering Integration Laboratory, Department of General Surgery, Affiliated Hospital of Southwest Jiaotong University, The Third People’s Hospital of Chengdu, Chengdu, China; 2 School of Basic Medical Sciences, Southwest Medical University, Luzhou, China; 3 Institute of Biomedical Engineering, College of Medicine, Southwest Jiaotong University, Chengdu, China; 4 Key Laboratory of Advanced Technologies of Materials, Ministry of Education, School of Materials Science and Engineering, Southwest Jiaotong University, Chengdu, China

**Keywords:** endothelial dysfunction, fibrosis, gut–liver axis, liver sinusoidal endothelial cells, metabolic dysfunction–associated fatty liver disease, vascular microenvironment

## Abstract

Metabolic dysfunction–associated fatty liver disease (MAFLD) is the leading cause of chronic liver disease worldwide and is increasingly recognized as a multisystem disorder driven by intertwined metabolic and vascular dysfunction. Beyond the classical features of steatosis, inflammation, and fibrosis, emerging evidence suggests that disruption of the hepatic vascular microenvironment may contribute to MAFLD progression, although its temporal relationship with metabolic injury requires further validation. The liver sinusoid constitutes a highly specialized vascular niche in which liver sinusoidal endothelial cells (LSECs) function as metabolic gatekeepers and mechanosensors, integrating blood flow, shear stress, and nutrient exchange to maintain hepatic homeostasis. In MAFLD, endothelial metabolic reprogramming, reduced nitric oxide bioavailability, and sinusoidal microcirculatory impairment collectively disrupt vascular regulation, thereby promoting lipotoxicity, inflammatory activation, and tissue hypoxia. These processes may be further amplified by multicellular crosstalk, wherein immune cells exacerbate endothelial dysfunction, while hepatic stellate cells (HSCs) promote extracellular matrix deposition, vascular stiffening, and increased sinusoidal resistance. In this review, we synthesize current evidence from a vascular microenvironment perspective and propose a stage-associated conceptual framework encompassing early endothelial stress, immune–endothelial amplification, and fibrotic remodeling. We also discuss emerging therapeutic strategies targeting endothelial metabolism, microcirculatory function, and vascular resistance. By integrating metabolic, inflammatory, and biomechanical processes, this review highlights the hepatic microvasculature as a candidate regulatory hub warranting prospective evaluation as a translational target in MAFLD.

## 1 Introduction

### 1.1 Definition and diagnostic criteria of MAFLD

Compared with the previous exclusion-based NAFLD definition, MAFLD adopts a positive diagnostic framework centered on metabolic dysfunction (1). It does not exclude concurrent chronic liver diseases, such as viral hepatitis or alcohol-related liver disease, thereby better reflecting real-world clinical complexity (2–4). This approach may improve the identification of individuals with metabolic risk and facilitate risk stratification, particularly for patients with significant fibrosis (5–7), while the updated terminology may enhance disease awareness and communication among patients and clinicians (4, 8–10).

This review primarily adopts the MAFLD definition, which emphasizes a positive diagnosis based on hepatic steatosis in the presence of metabolic dysfunction. We acknowledge the 2023 multi-society Delphi consensus that introduced the term metabolic dysfunction-associated steatotic liver disease (MASLD) and the corresponding steatohepatitis nomenclature (MASH). Where epidemiological estimates are derived from earlier NAFLD-defined cohorts, this is noted. In Section 4, the term MASH is used when referring to trial populations defined by histological criteria consistent with current clinical trial practice.

### 1.2 Epidemiology and comorbidities

MAFLD has become the most prevalent chronic liver disease worldwide, affecting approximately one-third of the adult population, with prevalence continuing to rise in parallel with the global increase in obesity and metabolic risk factors (11, 12). This upward trend is expected to continue, with projections from NAFLD-based epidemiological models suggesting that the global prevalence of fatty liver disease may exceed 50% by 2040 (approximately 55.7%) (13). Given the progressive nature of MAFLD, the increasing disease burden is expected to further amplify the clinical burden associated with advanced liver disease and its complications (1, 14–16). Liver-related mortality has correspondingly risen, imposing substantial medical and socioeconomic burdens (17–19).

Notably, MAFLD extends beyond the liver and is closely associated with systemic metabolic and vascular comorbidities, particularly cardiovascular disease and chronic kidney disease, highlighting its substantial extrahepatic disease burden (20–22). These associations underscore the close interplay between hepatic metabolism and systemic vascular health, suggesting that alterations within the hepatic circulation may contribute not only to intrahepatic injury but also to broader cardiometabolic risk.

### 1.3 The hepatic vascular microenvironment as an early regulatory hub in MAFLD

Beyond hepatocellular lipid accumulation, accumulating evidence suggests that alterations in the hepatic vascular microenvironment occur early during MAFLD progression and may function as important contributors to disease development (23, 24). The hepatic sinusoid is increasingly recognized as a dynamic regulatory niche rather than a passive conduit for blood flow (25, 26), where hemodynamic cues, endothelial metabolism, and multicellular signaling are tightly integrated to coordinate hepatic homeostasis and disease progression (24, 26–28). Within this specialized microenvironment, LSECs act as mechanosensors and metabolic gatekeepers that couple shear stress, oxygen delivery, and nutrient exchange to parenchymal and immune cell responses (24, 26, 27).

Disruption of sinusoidal perfusion, nitric oxide bioavailability, and flow-dependent signaling has been increasingly associated with steatosis, inflammation, and fibrogenesis. Emerging evidence suggests that microcirculatory dysfunction may contribute to disease progression through extensive interactions with metabolic, inflammatory, and fibrogenic pathways rather than functioning solely as a downstream manifestation of liver injury (23, 28, 29).

### 1.4 Scope and objectives of this review

In this review, we summarize current evidence on the hepatic vascular microenvironment in MAFLD. We discuss how alterations in sinusoidal structure, endothelial function, and multicellular communication influence metabolic regulation, inflammatory responses, and fibrotic remodeling during disease progression.

To provide a mechanistic perspective, the review is organized around a stage-associated conceptual framework of vascular events, encompassing early endothelial dysfunction, immune–vascular inflammatory amplification, and fibrogenic remodeling. By integrating these processes within a unified microcirculatory framework, we aim to clarify how vascular disturbances actively shape MAFLD pathogenesis and to highlight potential translational opportunities for improving diagnosis and therapeutic intervention.

While previous reviews have comprehensively addressed the biology of liver sinusoidal endothelial cells and their roles in chronic liver disease (24–28, 70), the present review specifically integrates the hepatic vascular microenvironment into a stage-associated conceptual framework for MAFLD. We further link this framework to the gut–liver–heart axis and critically examine the current translational gap—namely, that most clinically advanced MASH therapies lack dedicated vascular endpoints. In doing so, we aim to clarify both the mechanistic contribution of the microvasculature and its potential as a therapeutic and biomarker target.

This narrative review was informed by a literature search of PubMed/MEDLINE, Web of Science, and Scopus databases, covering publications from January 2000 to June 2026. Search terms included combinations of “MAFLD”, “MASLD”, “NAFLD”, “MASH”, “liver sinusoidal endothelial cells”, “LSEC”, “hepatic microcirculation”, “endothelial dysfunction”, “sinusoidal capillarization”, “portal hypertension”, and related vascular and therapeutic keywords. Priority was given to original research, high-quality reviews, and clinical trials relevant to the hepatic vascular microenvironment. Reference lists of key articles were also manually screened. Given the narrative nature of this review, formal systematic review protocols (e.g., PRISMA) were not applied.

## 2 Pathophysiological basis of MAFLD and vascular microenvironment

### 2.1 Pathogenesis

The pathogenesis of MAFLD is multifactorial, evolving from the traditional “two-hit hypothesis”, which centers on lipid accumulation and oxidative stress, to a more comprehensive “multiple parallel hits” model. This contemporary framework emphasizes the synergistic contributions of key pathogenic drivers, including insulin resistance, oxidative stress, chronic inflammation, and genetic susceptibility, thereby reflecting the complex etiology of MAFLD beyond a singular lipotoxic mechanism (30, 31).

Within this context, the hepatic vascular microenvironment serves as a central integrative platform for metabolic and immune regulation (32), coordinating intercellular crosstalk within the sinusoidal niche (28). This specialized niche comprises LSECs, hepatocytes, Kupffer cells (KCs), HSCs, and bile duct epithelial cells (BECs) (33, 34). Rather than acting as an isolated factor, the vascular microenvironment coordinates the effects of these pathogenic drivers by mediating intercellular crosstalk, hemodynamic regulation, and inflammatory signaling.

Functional impairments within these cellular components not only promote intrahepatic steatosis and fibrogenesis but also contribute to systemic vascular dysfunction through the “gut–liver–heart axis” (35, 36). This integrative framework provides a mechanistic basis for understanding how metabolic stress is translated into coordinated vascular and cellular responses during MAFLD progression.

Insulin resistance (IR) remains the principal pathogenic driver of MAFLD and exerts its effects within the hepatic vascular microenvironment through coordinated alterations across multiple cell types. Diminished insulin sensitivity in hepatic, adipose, and skeletal muscle tissues disrupts systemic glucose and lipid homeostasis (37).

In hepatocytes, impaired insulin signaling is associated with reduced tyrosine phosphorylation of insulin receptor substrate (IRS), thereby suppressing the phosphoinositide 3-kinase (PI3K)/Akt pathway, which may disrupt intracellular metabolic homeostasis and contribute to the accumulation and release of lipotoxic mediators into the sinusoidal microenvironment (37, 38). This has been shown to enhance *de novo* lipogenesis via activation of sterol regulatory element-binding protein 1c (SREBP-1c) (39), leading to upregulation of fatty acid synthase (FASN) and acetyl-CoA carboxylase (ACC) (40, 41), while also contributing to the regulation of gluconeogenic pathways and being associated with reduced fatty acid β-oxidation. These metabolic alterations contribute to intracellular lipid accumulation, thereby reinforcing lipotoxic stress within the hepatic vascular microenvironment.

Oxidative stress and inflammation serve as pivotal factors that exacerbate MAFLD progression within the hepatic vascular microenvironment (42, 43). In hepatocytes, lipid accumulation induces mitochondrial dysfunction and endoplasmic reticulum stress, leading to excessive generation of reactive oxygen species (ROS). Elevated ROS levels not only inflict direct cellular damage but also promote the formation of lipid peroxidation products such as malondialdehyde (MDA) (44).

Experimental and mechanistic studies suggest that these oxidative stress signals extend beyond hepatocytes to influence neighboring cells within the sinusoidal niche. In LSECs, ROS accumulation may disrupt endothelial integrity, reduce nitric oxide (NO) bioavailability, and increase vascular permeability, thereby contributing to microcirculatory dysfunction (45). Meanwhile, oxidative stress activates KCs, promoting their polarization toward a proinflammatory M1 phenotype (46). Activated KCs have been shown to release proinflammatory cytokines, including tumor necrosis factor-alpha (TNF-α) and interleukin-6 (IL-6), as well as chemokines such as C–C motif chemokine ligand 2 (CCL2) and C–X–C motif chemokine ligand 10 (CXCL10), which facilitate monocyte recruitment and amplify intrahepatic inflammation (46–48).

Furthermore, ROS-mediated activation of key signaling pathways, including nuclear factor κB (NF-κB) and the NOD-like receptor family pyrin domain-containing 3 (NLRP3) inflammasome, reinforces inflammatory signaling across multiple cell types. Extracellular vesicles (EVs) released from injured hepatocytes may further propagate these signals within the vascular microenvironment, potentially contributing to systemic vascular complications (49).

Together, the interplay between oxidative stress and inflammation drives multicellular activation within the hepatic vascular niche, contributing to endothelial dysfunction, immune cell recruitment, and progressive microvascular injury.

The susceptibility to MAFLD is further influenced by the interplay between genetic predisposition and environmental factors (50), which may modulate cellular responses within the hepatic vascular microenvironment (24, 32). Genetic variants, particularly patatin-like phospholipase domain-containing protein 3 (PNPLA3) and 17β-hydroxysteroid dehydrogenase type 13 (HSD17B13), have been identified as important determinants of disease susceptibility and progression (51, 52).

The PNPLA3 I148M variant promotes intracellular lipid retention and increases hepatic triglyceride content, thereby enhancing lipotoxic stress within the sinusoidal microenvironment and facilitating progression to steatohepatitis and fibrosis (53–56). Other susceptibility variants, including transmembrane 6 superfamily member 2 (TM6SF2) (which impairs very-low-density lipoprotein (VLDL) secretion and promotes hepatic lipid retention) and membrane bound O-acyltransferase domain containing 7 (MBOAT7) (which affects phospholipid remodeling), have also been associated with altered hepatic lipid handling and disease progression (57, 58). Conversely, loss-of-function variants in HSD17B13 exert protective effects by attenuating inflammatory and fibrogenic responses and are associated with reduced risks of MASH progression, fibrosis progression, and hepatocellular carcinoma (59–61).

Collectively, these genetic factors primarily influence hepatocyte metabolism and stress responses and may indirectly reshape the hepatic vascular microenvironment through alterations in lipid handling, inflammatory activation, and multicellular communication.

### 2.2 Pathological characteristics of MAFLD

#### 2.2.1 Histopathological progression and systemic complications of MAFLD

##### 2.2.1.1 Hepatic steatosis

In the early stage of MAFLD, the predominant histological feature is hepatic steatosis, characterized by lipid droplet accumulation in ≥5% of hepatocytes, typically exhibiting a macrovesicular pattern, with or without mild, nonspecific inflammation. Imaging and liver biopsy commonly reveal these changes. As the disease progresses, some patients develop lobular inflammation and hepatocellular ballooning, indicating transition toward metabolic dysfunction–associated steatohepatitis (MASH) (62).

##### 2.2.1.2 Fibrosis

Hepatic fibrosis represents a central pathological hallmark in the progression of MAFLD. The disease typically evolves from simple steatosis to MASH, and subsequently to advanced fibrosis or cirrhosis (31, 63). The fibrotic process results from chronic injury and aberrant tissue repair, leading to excessive extracellular matrix (ECM) deposition and architectural remodeling of hepatic parenchyma (31, 64, 65). Persistent fibrogenesis disrupts microvascular homeostasis, aggravating intrahepatic hypoxia and further amplifying inflammation—a self-perpetuating loop that links fibrosis to vascular dysfunction (33, 66).

##### 2.2.1.3 Cardiovascular complications

As discussed in Section 1.2, MAFLD is closely associated with increased cardiovascular risk and is increasingly recognized as a systemic metabolic–vascular disorder (1, 67).

#### 2.2.2 Integrative perspective

Overall, MAFLD involves interconnected metabolic, inflammatory, fibrotic, and systemic vascular alterations. Within this complex network, dysfunction of the hepatic vascular microenvironment may serve as an important pathogenic link connecting intrahepatic injury with systemic metabolic and cardiovascular disturbances. This integrative perspective provides a mechanistic basis for understanding the cellular and molecular interactions discussed in the subsequent sections.

#### 2.2.3 Vascular microenvironment-related pathology: sinusoidal microcirculatory disturbance

Vascular microenvironmental alterations play a pivotal role in the onset and progression of MAFLD, serving as a central link between intrahepatic injury and systemic metabolic dysfunction. These integrated gut–liver–heart interactions and their vascular consequences are illustrated within the systemic framework shown in **Figure 1**.

**Figure 1:**
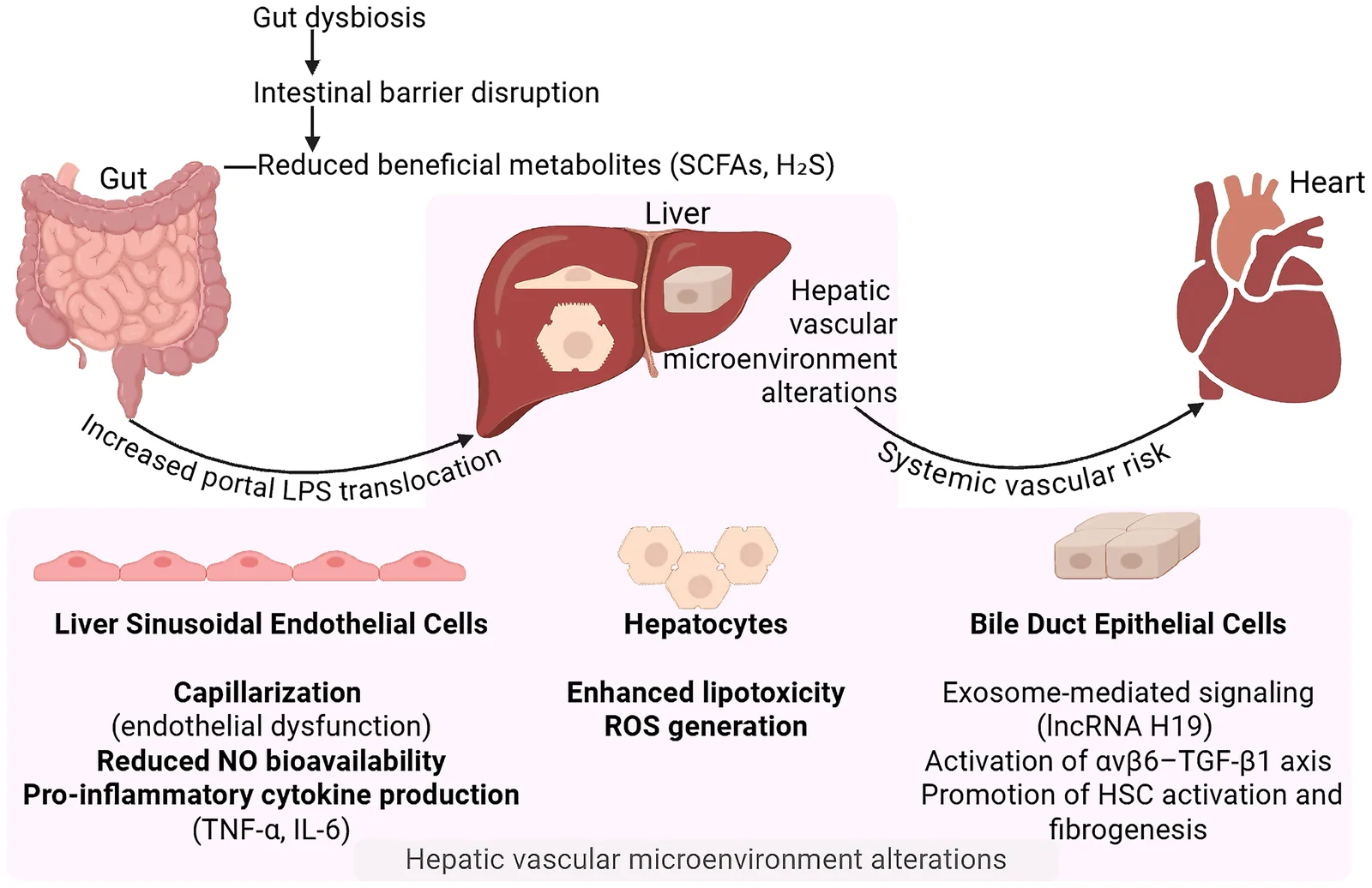
Gut–liver–heart axis and vascular microenvironment alterations in metabolic dysfunction–associated fatty liver disease (MAFLD). Gut dysbiosis and intestinal barrier disruption increase portal lipopolysaccharide (LPS) translocation and reduce beneficial metabolites such as short-chain fatty acids (SCFAs) and hydrogen sulfide (H_2_S). In the liver, LPS and hepatocyte lipotoxicity induce liver sinusoidal endothelial cell (LSEC) dysfunction (capillarization, reduced nitric oxide (NO) bioavailability) and inflammatory activation, which together with hepatic stellate cell (HSC) activation disrupt the hepatic vascular microenvironment and contribute to systemic vascular risk along the gut–liver–heart axis.

In the early stages of MAFLD, sinusoidal microcirculatory disturbance represents one of the earliest indicators of portal hypertension. The hepatic sinusoid, a specialized low-pressure and low-shear capillary network, exhibits increased intrahepatic vascular resistance—a process that involves both functional dysregulation and structural remodeling (68, 69).

LSECs generate NO via endothelial nitric oxide synthase (eNOS) to maintain vasodilation and microcirculatory homeostasis. In experimental models and human liver samples, LSECs exhibit features of capillarization, characterized by fenestrae loss, abnormal basement membrane deposition, and decreased eNOS activity, leading to reduced NO bioavailability (70). This endothelial dysfunction is accompanied by increased release of vasoconstrictors such as endothelin-1 (ET-1) and thromboxane A_2_, further aggravating sinusoidal constriction (26, 71). Meanwhile, experimental studies in fatty liver models suggest that hepatocyte ballooning and lipid accumulation are associated with altered sinusoidal architecture and impaired hepatic microcirculation. In rodent models of steatohepatitis, histological and biomechanical analyses have demonstrated increased hepatic tissue stiffness and structural distortion, which may contribute to reduced sinusoidal perfusion and localized hypoxia through mechanical and hemodynamic mechanisms (72, 73). In addition, activation of NOX-dependent oxidative pathways in LSECs has been shown to increase ROS production, impair endothelial function, and reduce NO bioavailability in NAFLD models (45), suggesting a potential mechanism contributing to sinusoidal microcirculatory dysfunction in MAFLD. During inflammatory progression, hepatocellular swelling and inflammatory remodeling may further impair sinusoidal architecture and microcirculatory flow (72, 73). Experimental studies indicate that reduced NO bioavailability promotes HSC contractile responses and extracellular matrix deposition, thereby contributing to sinusoidal constriction and the progression of microvascular dysfunction (74–76).

Experimental evidence supports this pathophysiological loop. Fecal microbiota transplantation (FMT) from healthy controls into high-fat diet–induced MAFLD mice alleviates portal pressure and restores protein kinase B (Akt)/eNOS signaling, suggesting that gut microbiota regulate endothelial homeostasis via the bile acid–farnesoid X receptor (FXR) pathway (77).

Gut–liver axis dysregulation further amplifies these vascular changes. Experimental studies suggest that microbiota-derived short-chain fatty acids (SCFAs) may influence endothelial homeostasis by enhancing nitric oxide (NO) bioavailability and reducing oxidative stress (77, 78), while endogenous hydrogen sulfide (H_2_S), produced by cystathionine γ-lyase (CSE), may regulate oxidative stress and vascular tone in experimental models of NAFLD (79, 80). Nuclear receptors FXR and G protein–coupled bile acid receptor 1 (GPBAR1) are known to upregulate CSE and other enzymes involved in H_2_S synthesis (81, 82). However, reduced microbial diversity in MAFLD leads to impaired H_2_S production, exacerbating endothelial dysfunction and vascular constriction (68). Disruption of the intestinal barrier also allows bacterial membrane components—such as phosphatidylglycerol (PG) and phosphatidylethanolamine (PE)—to translocate into the portal circulation, directly activating KCs and worsening microcirculatory inflammation (83). Furthermore, dysbiosis-induced bile acid imbalance establishes a vicious cycle of endotoxemia–inflammation–fibrosis that links intrahepatic lipid accumulation with systemic vascular injury via the “gut–liver–heart axis” described earlier (35, 84).

Collectively, these mechanisms illustrate a bidirectional regulatory loop between sinusoidal microcirculatory disturbance and gut–liver axis dysfunction, jointly driving the transition of MAFLD from localized hepatic steatosis to systemic metabolic disorder.

## 3 Stage-associated conceptual framework of the hepatic vascular microenvironment in MAFLD

MAFLD is widely regarded as a multifactorial disorder driven by multiple parallel and interacting pathogenic processes rather than a strictly linear cascade (31). Nevertheless, vascular alterations occur throughout disease progression and exhibit extensive crosstalk with metabolic, inflammatory, and fibrogenic pathways. Therefore, the stage-associated conceptual framework presented below highlights vascular remodeling as an organizing axis within the broader multiple-hit paradigm.

Within the hepatic vascular microenvironment, LSECs function as metabolic gatekeepers and mechanosensors that dynamically couple blood flow, shear stress, and nutrient flux to parenchymal metabolism and inflammatory tone (24, 32). In MAFLD, disturbances in sinusoidal perfusion and endothelial metabolism are increasingly recognized as important features of disease progression and contribute to the reshaping of intercellular communication within the hepatic niche (23, 70). Through flow-dependent signaling, NO bioavailability, and barrier remodeling, LSECs interact extensively with hepatocytes, KCs, HSCs, and BECs, forming interconnected regulatory networks that link metabolic stress, inflammation, microcirculatory dysfunction, and fibrogenesis. These alterations evolve dynamically during disease progression and reflect stage-associated remodeling of the hepatic vascular niche.

To better integrate these complex interactions, we propose a stage-associated conceptual framework centered on the hepatic vascular microenvironment. **Figure 2** summarizes the key cellular and signaling interactions among LSECs, hepatocytes, Kupffer cells, and HSCs across disease stages, while the structural and mechanical consequences of these interactions are detailed in **Figure 3**.

**Figure 2:**
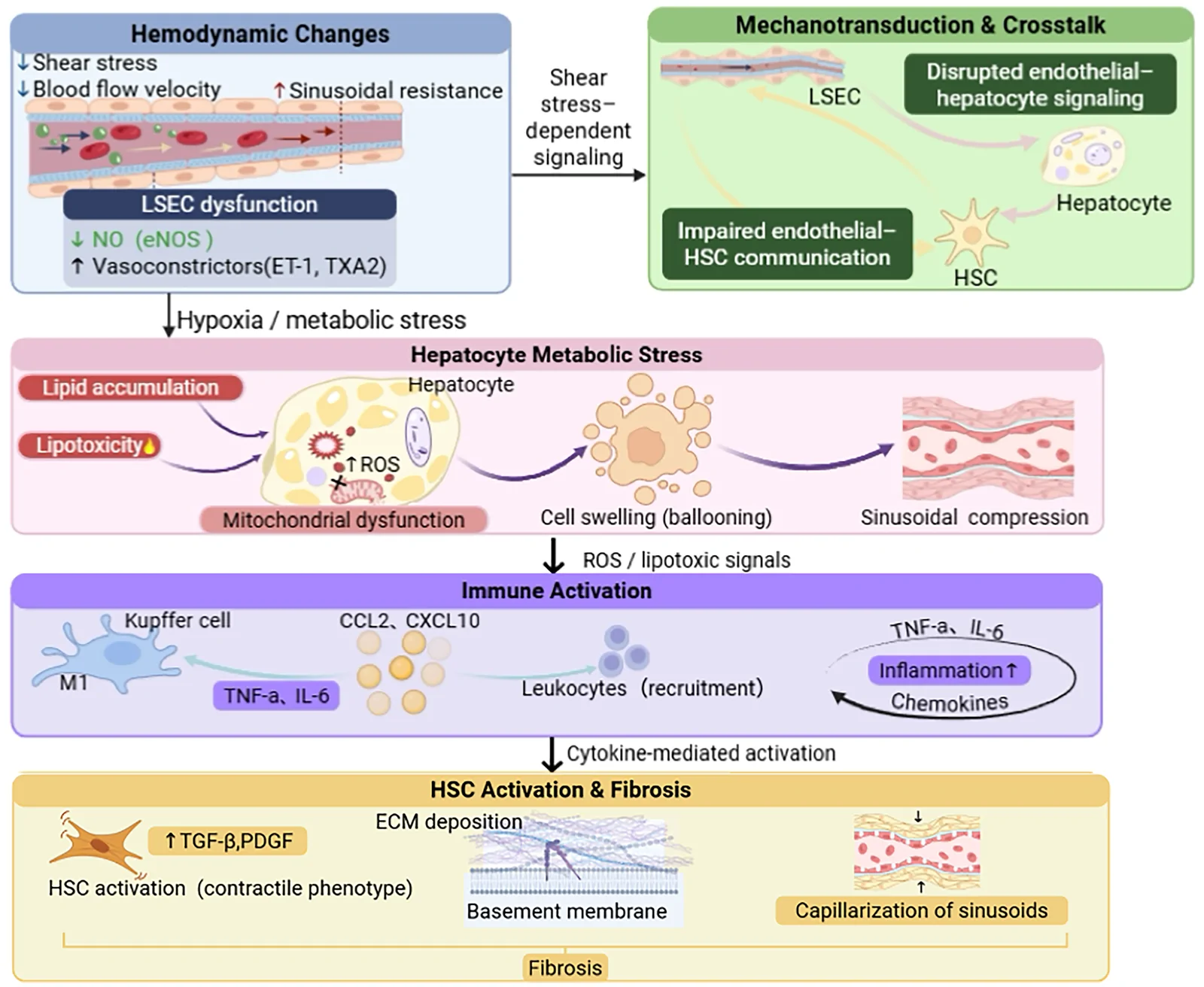
Stage-associated cellular and signaling interactions within the hepatic vascular microenvironment in metabolic dysfunction–associated fatty liver disease (MAFLD). Hemodynamic disturbances and liver sinusoidal endothelial cell (LSEC) dysfunction (impaired nitric oxide (NO) bioavailability) are linked to hepatocyte lipotoxicity, Kupffer cell activation, and subsequent hepatic stellate cell (HSC) activation via transforming growth factor-β (TGF-β) and platelet-derived growth factor (PDGF). These interconnected processes illustrate how the vascular niche interacts with metabolic, immune, and fibrogenic pathways during disease progression.

**Figure 3:**
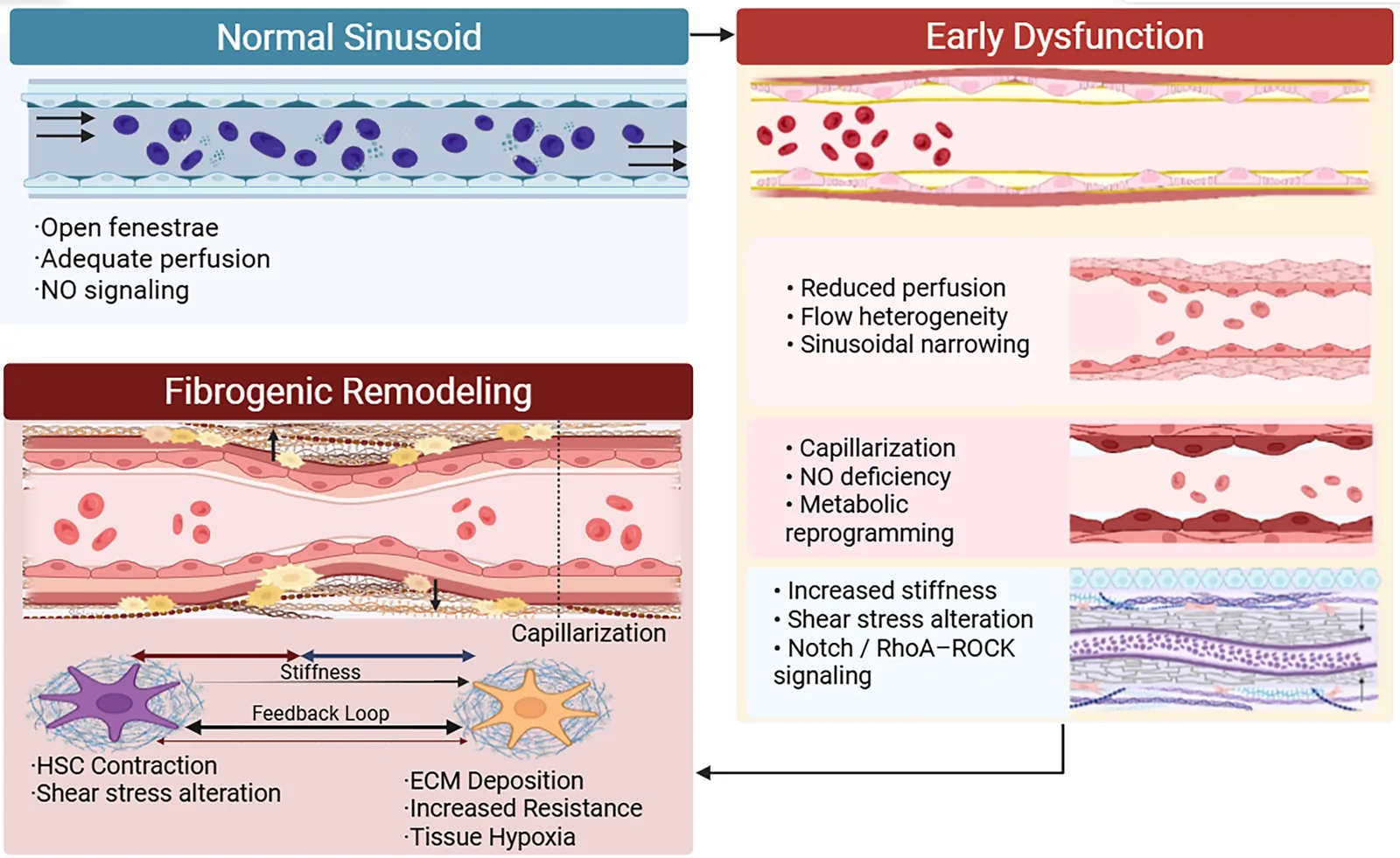
Progressive sinusoidal remodeling and mechanical feedback in metabolic dysfunction–associated fatty liver disease (MAFLD). Early changes include reduced perfusion and liver sinusoidal endothelial cell (LSEC) capillarization. With progression, matrix stiffness and hepatic stellate cell (HSC) contraction create a positive feedback loop that increases intrahepatic resistance, promotes hypoxia, and further impairs sinusoidal plasticity.

### 3.1 Early metabolic stress: LSEC dysfunction and sinusoidal microvascular remodeling

LSECs represent a central structural and functional component of the hepatic vascular microenvironment, accounting for approximately 15–20% of total liver cells (85). Under physiological conditions, LSECs possess characteristic fenestrae with a diameter range of 50–200 nm and lack a continuous basement membrane, thereby facilitating efficient exchange of lipids and metabolites between blood and hepatocytes and maintaining metabolic and immune homeostasis (26, 86–89).

Building upon these structural and functional characteristics, LSEC dysfunction is increasingly recognized as an important feature of MAFLD progression that contributes to alterations in the hepatic vascular microenvironment and is closely associated with metabolic, inflammatory, and fibrogenic responses.

As early as 1997, Shah et al. demonstrated that LSECs are the predominant source of NO in the liver, which is essential for maintaining sinusoidal tone and hepatic microcirculatory function (90). In MAFLD, LSECs undergo profound phenotypic and functional alterations, resulting in impaired sinusoidal exchange capacity, endothelial dysfunction, and disturbed lipid transport (24, 70). Progressive LSEC capillarization, characterized by loss of fenestrae and deposition of a continuous basement membrane, represents a hallmark of sinusoidal endothelial dysfunction during chronic liver disease progression (24, 27, 91). In addition, Notch signaling activation has been shown to impair LSEC function by suppressing eNOS expression and NO production during NASH progression (92). This reduction in NO bioavailability disrupts vascular tone and hepatic lipid homeostasis by enhancing lipogenesis and limiting fatty acid oxidation, thereby accelerating hepatic lipid accumulation and disease progression (93).

Oxidized low-density lipoprotein (oxLDL) can activate lectin-like oxidized LDL receptor-1 (LOX-1) signaling in LSECs, promoting oxidative stress and endothelial dysfunction, thereby contributing to sinusoidal capillarization during metabolic stress (94). This structural remodeling compromises the selective permeability of LSECs, restricting the trans-sinusoidal transfer of triglycerides, cholesterol, and chylomicron remnants, which further promotes intrahepatic lipid retention (70, 95). Moreover, elevated circulating lipid species—including cholesterol, triglycerides, LDL, and chylomicron remnants—stimulate *de novo* lipogenesis in hepatocytes, amplifying steatosis and metabolic imbalance (96, 97).

Beyond structural remodeling, accumulating evidence suggests that early disturbances in sinusoidal perfusion and shear stress accompany the initial metabolic alterations in MAFLD and may contribute to subsequent inflammatory and fibrotic remodeling (23). Reduced sinusoidal perfusion and microcirculatory disturbances disrupt shear stress–dependent signaling in LSECs (23, 93, 98), contributing to impaired eNOS activation and diminished NO bioavailability (70, 98). These hemodynamic alterations, together with capillarization, exacerbate regional hypoxia and create a microenvironment permissive for lipid retention and fibrogenic signaling during MAFLD progression (70, 99, 100).

At the metabolic level, LSECs undergo early endothelial metabolic reprogramming under lipotoxic stress (101, 102). Under physiological conditions, endothelial cells rely predominantly on glycolysis to meet their energetic demands while preserving oxygen delivery to parenchymal cells (103, 104).

In MAFLD, metabolic overload disrupts endothelial redox homeostasis and eNOS coupling through impaired reduced nicotinamide adenine dinucleotide phosphate (NADPH) availability and suppression of fatty acid oxidation, leading to excessive ROS accumulation and reduced NO bioavailability (98, 105). This metabolic imbalance not only compromises endothelial barrier integrity but also promotes endothelial injury and progressive microvascular dysfunction (26, 27).

Beyond metabolic regulation, LSECs play a pivotal role in hepatic immune tolerance. Under homeostatic conditions, LSECs suppress excessive T-cell activation through both direct cell contact and soluble immunomodulatory mediators, maintaining a tolerogenic intrahepatic immune milieu (29, 106). In MAFLD, however, capillarized LSECs lose this tolerogenic phenotype and instead upregulate pro-inflammatory cytokines, including TNF-α, IL-6, interleukin-1β (IL-1β), and CCL2, as well as adhesion molecules such as vascular cell adhesion molecule-1 (VCAM-1) and intercellular adhesion molecule-1 (ICAM-1) (107, 108). These changes promote recruitment and infiltration of macrophages and monocytes into hepatic tissue, thereby amplifying local inflammation and disrupting immune homeostasis (107, 109, 110).

Multiple molecular pathways contribute to LSEC dysfunction in MAFLD. Early upregulation of NADPH oxidase 1 (NOX1) decreases NO bioavailability, leading to endothelial dysfunction (45). Impaired autophagy enhances the expression of CCL2, C–C motif chemokine ligand 5 (CCL5), and VCAM-1, promoting monocyte recruitment and accelerating inflammation and fibrogenesis (107, 108, 111). Furthermore, shear stress–induced activation of the Notch–Sirtuin 1–p21/p16 signaling axis promotes LSEC senescence, disrupting sinusoidal homeostasis, impairing hepatocyte regeneration, and increasing fibrogenic susceptibility (112, 113).

Normally, LSECs maintain hepatic quiescence by restraining HSCs activation; however, capillarized LSECs lose this regulatory capacity, resulting in sustained HSCs activation and progressive fibrosis (114, 115). Notably, Hedgehog signaling contributes to LSEC capillarization, and inhibition of this pathway can partially reverse capillarization-associated phenotypic changes and preserve a more differentiated LSEC state (116).

Collectively, LSEC dysfunction actively contributes to metabolic imbalance, inflammation, and fibrogenesis through structural, metabolic, immune, and hemodynamic alterations, supporting its role as a key regulatory interface in the hepatic vascular microenvironment.

### 3.2 Inflammatory amplification: immune–endothelial crosstalk and vascular activation

Following early sinusoidal dysfunction and endothelial metabolic impairment, persistent lipid overload progressively converts the hepatic vascular niche from a metabolically stressed state into an inflammatory microenvironment. During this transition, hepatocyte-derived danger signals and immune activation act as secondary drivers that amplify endothelial injury and microvascular instability.

Hepatocytes, the principal parenchymal cells responsible for metabolic homeostasis, are the earliest targets of lipid excess. In MAFLD, triglyceride accumulation induces steatosis, insulin resistance, and oxidative stress, initiating lipotoxic signaling cascades that extend beyond hepatocytes themselves (31, 37). Under metabolic stress, hepatocytes release paracrine mediators that perturb the vascular niche, establishing a self-reinforcing “lipotoxicity–inflammation–vascular injury” loop.

EVs constitute a key mechanism of this intercellular communication. Hepatocyte-derived EVs enriched with inflammatory mediators, including miR-192-5p, activate macrophages (including KCs and monocyte-derived macrophages), promoting polarization toward a proinflammatory M1 phenotype (117).These cells subsequently activate NF-κB and mitogen-activated protein kinase (MAPK) signaling and release TNF-α and IL-6 (93, 118, 119). The resulting cytokine surge upregulates VCAM-1 and ICAM-1 on LSECs, thereby enhancing leukocyte adhesion and infiltration and shifting the sinusoidal endothelium from a tolerogenic to an inflammatory phenotype (120–123).

In parallel, lipid overload–induced mitochondrial dysfunction in hepatocytes generates excessive ROS, which disrupt mitochondrial membrane potential, impair adenosine triphosphate (ATP) synthesis, and disturb cellular energetics (124, 125). These ROS and oxidative mediators can further diffuse into the sinusoidal space, compromising endothelial integrity, increasing vascular permeability, and promoting inflammatory cell extravasation, thereby intensifying microvascular inflammation and fibrosis (45). Mitochondrial dysfunction thus represents a critical link between hepatocellular metabolic stress and vascular microenvironmental imbalance (124–126). Impaired mitophagy further exacerbates this process by promoting mitochondrial reactive oxygen species (mtROS) accumulation and NLRP3 inflammasome activation, thereby amplifying inflammatory and fibrogenic responses (127).

As resident hepatic macrophages, KCs serve as central amplifiers of this inflammatory cascade. Although they normally maintain immune tolerance, metabolic stress and endotoxemia rapidly drive KCs activation. Lipid overload upregulates scavenger receptors such as macrophage scavenger receptor 1 (MSR1), enhancing lipid uptake and activating the c-Jun N-terminal kinase (JNK) pathway to promote M1 polarization and ROS production (128, 129). Simultaneously, gut barrier dysfunction facilitates lipopolysaccharide (LPS) translocation into the portal circulation, where LPS engages toll-like receptor 4 (TLR4)/cluster of differentiation 14 (CD14) signaling on KCs, triggering NF-κB–dependent transcription and further TNF-α and IL-6 release (84, 130, 131).

KC-derived pro-inflammatory mediators contribute to LSEC activation and endothelial dysfunction. Inflammatory signaling promotes the expression of adhesion molecules, including VCAM-1 and ICAM-1, on LSECs, facilitating leukocyte adhesion and recruitment (132). This endothelial dysfunction is accompanied by impaired NO signaling and LSEC capillarization, which together disrupt sinusoidal vascular function and microvascular perfusion (27, 133). Meanwhile, chemokines such as CCL2 and CXCL10 recruit inflammatory monocytes and activate HSCs, thereby promoting progressive fibrogenesis (134). Chronic metabolic stress can alter KCs polarization through pathways involving signal transducer and activator of transcription 3 (STAT3) signaling, thereby contributing to inflammatory and fibrogenic responses in the liver (135, 136). Activated macrophage populations can further promote hepatic stellate cell activation and fibrogenesis, highlighting the important role of macrophage–HSC crosstalk in disease progression (137, 138). Single-cell and transcriptomic studies have further revealed substantial heterogeneity among hepatic macrophage populations, suggesting that distinct macrophage subsets may exert different functions during liver injury and disease progression (139).

Therapeutically, targeting inflammatory pathways such as the NLRP3 inflammasome or chemokine axes involving C–C chemokine receptor 2 (CCR2) and C–C chemokine receptor 5 (CCR5) has shown potential to attenuate hepatic inflammation and fibrotic remodeling in preclinical models and clinical studies (140, 141). However, although the dual CCR2/CCR5 antagonist cenicriviroc showed antifibrotic potential in the phase 2b CENTAUR trial (140), the subsequent phase 3 AURORA trial failed to demonstrate a significant improvement in liver fibrosis (142). These findings underscore the challenges of translating anti-inflammatory approaches into clinical benefit.

Collectively, hepatocyte lipotoxicity, EV-mediated immune activation, mitochondrial oxidative stress, and KC-driven inflammation form a coordinated amplification loop that progressively destabilizes the sinusoidal vascular niche. This stage represents the transition from isolated endothelial dysfunction to overt inflammatory remodeling, creating conditions that favor HSC activation, extracellular matrix deposition, and subsequent fibrotic progression.

### 3.3 Fibrogenic remodeling: loss of vascular plasticity and sinusoidal resistance

As MAFLD progresses from inflammatory activation to fibrogenesis, the hepatic vascular niche undergoes profound structural and mechanical remodeling characterized by excessive ECM deposition, sinusoidal narrowing, and loss of microvascular plasticity. While **Figure 2** outlines the cellular and signaling cascade leading to HSC activation, the progressive mechanical and structural remodeling of the sinusoid is illustrated in **Figure 3**. HSCs serve as major effector cells involved in this transition and functionally link metabolic stress, inflammation, and vascular remodeling.

In their quiescent state, HSCs store vitamin A; however, under oxidative and inflammatory stress they transdifferentiate into contractile myofibroblast-like cells characterized by increased collagen synthesis and ECM deposition (74, 143). Single-cell transcriptomic analyses have revealed substantial heterogeneity among HSC subpopulations, including proliferative and myofibroblast-like populations as well as subsets associated with immune responses and vascular remodeling (143).

Beyond matrix production, activated HSCs acquire smooth muscle–like contractile properties and respond to vasoactive mediators such as endothelin-1 and angiotensin II, which contribute to increased intrahepatic vascular resistance and narrowing of the sinusoidal lumen (74, 144–146). Functionally, activated HSCs contribute to the regulation of sinusoidal tone and intrahepatic vascular resistance (74). Consequently, both cellular contraction and matrix accumulation synergistically elevate sinusoidal resistance and impair hepatic perfusion.

Activated HSCs undergo metabolic reprogramming characterized by a Warburg-like shift toward aerobic glycolysis, with enhanced glycolytic activity and altered glucose metabolism (147, 148). This metabolic transition is associated with increased expression of glycolytic regulators, including glucose transporter 1 (GLUT1) and pyruvate kinase M2 (PKM2), while additional regulatory mechanisms have been discussed in recent reviews (149).Glutamine metabolism fuels this activation: glutaminase 1 (GLS1) converts glutamine to α-ketoglutarate to sustain the tricarboxylic acid(TCA) cycle and collagen synthesis, whereas reduced proline oxidase activity promotes proline accumulation for ECM production (150, 151). Clinically, glutaminase (GLS), glutamate dehydrogenase 1 (GLUD1), and glutamic-oxaloacetic transaminase 1 (GOT1) are upregulated in fibrotic livers, whereas proline oxidase (POX) is downregulated (151). Lipid metabolic remodeling, including autophagy-dependent degradation of retinyl esters and reduced peroxisome proliferator-activated receptor gamma (PPAR-γ) signaling, further stabilizes the activated phenotype (152, 153).

Progressive ECM deposition during fibrosis markedly increases hepatic tissue stiffness and disrupts mechanical homeostasis within the sinusoidal wall (154–156). Matrix stiffening activates mechanotransduction pathways, including Ras homolog family member A (RhoA)/Rho-associated coiled-coil containing protein kinase (ROCK) signaling, which enhance vascular contractility and contribute to elevated intrahepatic vascular resistance and portal pressure (157, 158). These mechanical alterations are therefore likely to impair normal sinusoidal deformability and microcirculatory regulation, further aggravating hepatic microvascular dysfunction.

Consistently, sinusoidal narrowing, capillarization, and progressive extracellular matrix accumulation collectively contribute to hepatic microcirculatory disturbances, including altered flow patterns and regional hypoperfusion. Such changes are associated with impaired oxygen exchange and localized hypoxia, which in turn can exacerbate lipotoxicity, inflammatory responses, and fibrogenic signaling in chronic liver disease (156, 159–161). These hemodynamic disturbances suggest that fibrosis may not merely represent a structural consequence but could also contribute to further microcirculatory impairment.

Vascular signaling pathways further shape HSCs behavior. Specifically, Hedgehog, transforming growth factor-β1 (TGF-β1), Ras, and cellular Myc (c-Myc) pathways regulate metabolic enzyme expression: Hedgehog inhibition reduces glutaminase (GLS) and restores POX, whereas TGF-β1 suppresses POX (151). The role of TGF-β1 in enhancing collagen deposition and promoting vascular remodeling during fibrogenesis is well-established across parenchymal organs (162). Oxidative and endoplasmic reticulum (ER) stress also sustain HSCs activation: NOX-derived ROS activate the TGF-β/SMAD axis to induce alpha-smooth muscle actin (α-SMA) and collagen type I alpha 1 chain (COL1A1), while ER stress mediated by protein kinase RNA-like endoplasmic reticulum kinase (PERK) and X-box binding protein 1 (XBP1) enhances autophagy and metabolic resilience (149, 163–165).

In parallel, BECs contribute to fibrogenic remodeling through exosome-mediated and integrin-dependent signaling. BEC-derived exosomes carrying long noncoding RNA H19 promote HSC proliferation and fibrogenic responses, including extracellular matrix deposition (166), while integrin αvβ6-mediated activation of latent TGF-β1 contributes to ductular reaction and hepatic fibrosis (167). Together, these mechanisms may couple ductular responses to progressive fibrogenic and vascular remodeling within the hepatic microenvironment.

Collectively, fibrogenic remodeling progressively reduces sinusoidal compliance and vascular plasticity, thereby contributing to increased intrahepatic vascular resistance and impaired microcirculatory regulation. These structural and mechanical changes, including matrix stiffening, sinusoidal narrowing, and loss of vascular plasticity, are summarized in **Figure 3**.

To summarize the stage-associated framework and the current evidence base supporting each phase, the main vascular and cellular events, predominant sources of evidence, and overall evidence levels are outlined in **Table 1**.

**Table 1:** Stage-associated vascular events in MAFLD and current evidence levels.

Stage	Key vascular/cellular events	Main evidence base	Evidence level
Early metabolic stress	LSEC capillarization, reduced NO bioavailability, endothelial metabolic reprogramming	Rodent models; limited human liver samples	Predominantly preclinical
Inflammatory amplification	Kupffer cell activation, leukocyte recruitment, upregulation of adhesion molecules	Rodent models; some human transcriptomic data	Predominantly preclinical
Fibrogenic remodeling	HSC activation, matrix stiffening, sinusoidal narrowing, increased intrahepatic resistance	Rodent models; observational findings in human fibrotic liver	Mixed (preclinical + observational)
Therapeutic modulation	Direct endothelial/hemodynamic effects vs secondary metabolic–histological effects	Experimental data (e.g., statins, selected PPAR agonists); clinical trials lacking dedicated vascular endpoints	Limited direct clinical vascular evidence

Evidence levels are summarized qualitatively based on the predominant study type. Most mechanistic insights into the hepatic vascular microenvironment in MAFLD currently derive from experimental models. Dedicated longitudinal human studies that incorporate vascular or hemodynamic endpoints remain scarce.

## 4 Therapeutic strategies targeting the hepatic vascular microenvironment

MAFLD, introduced as an alternative diagnostic framework to NAFLD, has become one of the most prevalent chronic progressive liver diseases worldwide, affecting nearly one-third of adults globally (11, 12, 17). Recent epidemiological analyses using the updated MASLD framework further highlight the substantial burden of metabolic dysfunction-associated steatotic liver disease in China (168). In parallel with this global trend, MAFLD has become highly prevalent in China, with large-scale epidemiological studies reporting that approximately one-third of adults are affected (168, 169). The increasing disease burden is closely associated with rapid urbanization, sedentary lifestyles, and dietary transitions toward high-calorie diets (170). The rising clinical and socioeconomic burden of MAFLD underscores the urgent need for effective prevention, early screening, and targeted therapeutic strategies.

Thus, early screening and comprehensive management are of paramount importance. However, no universally approved disease-modifying therapy is currently available for all patients with MAFLD. Lifestyle modification, including improvement of dietary quality and increased physical activity, remains the cornerstone of management (171–174). Pharmacological therapies may be considered for selected patients with persistent metabolic risk factors, progressive liver injury, or advanced disease according to individual clinical characteristics and current guidelines (175). For patients with decompensated cirrhosis or end-stage liver disease, liver transplantation remains an established therapeutic option and should be considered according to disease severity and transplant eligibility criteria (176).

Given the central role of vascular dysfunction and fibrogenic remodeling in MAFLD progression, the hepatic vascular microenvironment represents a conceptually attractive therapeutic target. Current interventions act at multiple biological levels. A limited number of approaches (e.g., statins and selected PPAR agonists) have experimental or translational evidence of direct effects on endothelial function or hemodynamics, whereas the majority of clinically advanced agents primarily improve metabolic and histological endpoints and may only secondarily influence the vascular niche. These distinctions are summarized in **Figure 4** and discussed below.

**Figure 4:**
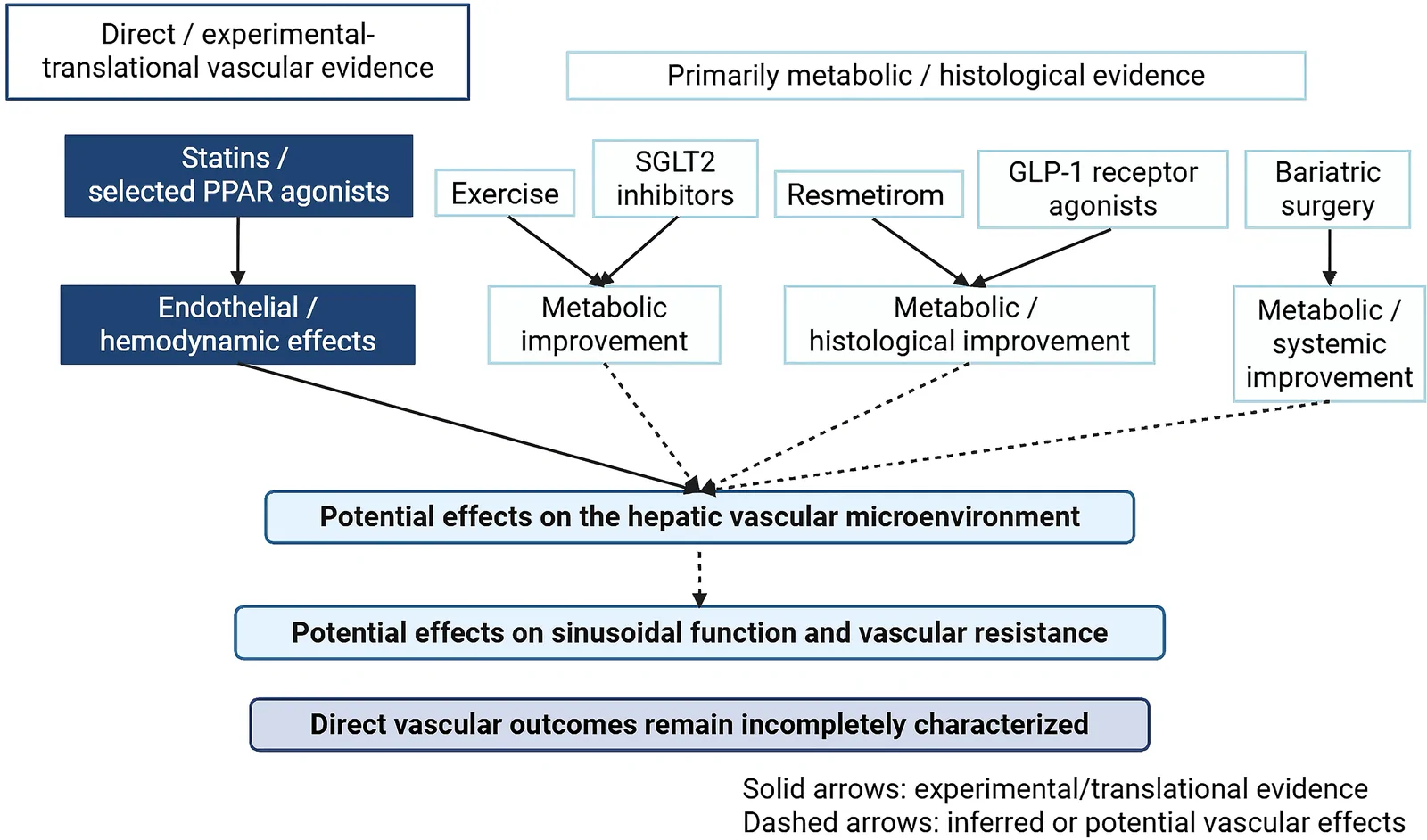
Therapeutic approaches with potential effects on the hepatic vascular microenvironment in metabolic dysfunction–associated fatty liver disease (MAFLD). Certain agents (statins and selected peroxisome proliferator-activated receptor [PPAR] agonists) have experimental or translational evidence supporting effects on endothelial function or hemodynamics (solid arrows). In contrast, lifestyle interventions (exercise) and agents such as resmetirom, glucagon-like peptide-1 (GLP-1) receptor agonists, sodium-glucose cotransporter 2 (SGLT2) inhibitors, and bariatric surgery primarily improve metabolic, histological, or systemic endpoints; any benefit on the hepatic vascular microenvironment is currently inferred (dashed arrows) and has not been directly measured as a prespecified endpoint in pivotal trials. Direct vascular outcomes therefore remain incompletely characterized. The figure illustrates these distinct pathways rather than proven causal restoration of the vascular niche.

### 4.1 Modulation of endothelial dysfunction and sinusoidal homeostasis

Lifestyle interventions remain the first-line approach for MAFLD and exert substantial benefits on the hepatic vascular niche.

Dietary patterns rich in saturated fats and refined carbohydrates promote oxidative stress and inflammatory responses that contribute to endothelial dysfunction and may facilitate sinusoidal capillarization in MAFLD (177, 178). In contrast, ω-3 polyunsaturated fatty acids and dietary fiber have been shown to suppress NF-κB–mediated inflammation and reduce TNF-α production, which may help attenuate endothelial inflammatory stress and support sinusoidal homeostasis (179–181). Modulation of the gut microbiota further reduces endotoxemia and protects portal endothelial integrity (35, 84).

Regular aerobic exercise not only decreases hepatic fat accumulation but also improves vascular function. Exercise training has been shown to reverse endothelial dysfunction in patients with NAFLD and to preserve hepatic eNOS activation in experimental models, which may contribute to maintenance of hepatic microcirculatory homeostasis (182, 183). These endothelial benefits may occur independently of weight loss and contribute to normalization of vascular tone and metabolic parameters (179, 184–186).

Statins have been reported to activate Kruppel-like factor 2 (KLF2)-mediated endothelial protective pathways and improve endothelial function (187). Although their specific effects on MAFLD-associated hepatic sinusoidal dysfunction remain to be fully defined in clinical studies, experimental data support a direct endothelial action. Peroxisome proliferator-activated receptor alpha (PPARα)-related and pan-PPAR therapies (including lanifibranor) have demonstrated improvements in fibrosis, endothelial abnormalities, and portal pressure in preclinical models of liver disease (188, 189), and recent work has further characterized LSEC phenotypic changes under lanifibranor (190), providing one of the clearer examples of agents with measurable hemodynamic readouts. Together, these findings highlight endothelial metabolism and NO signaling as potential therapeutic targets within the hepatic vascular microenvironment.

### 4.2 Modulation of immune–vascular inflammation

Chronic inflammation represents a second major contributor to vascular injury in MAFLD. Strategies that attenuate inflammatory signaling may therefore indirectly preserve endothelial integrity.

Polyphenolic compounds have shown promise in this regard. Resveratrol has been reported to exert endothelial protective effects through AMP-activated protein kinase (AMPK)-related signaling activation and reduction of oxidative stress (191, 192). In addition, sirtuin 1 (SIRT1)-dependent activation of peroxisome proliferator-activated receptor gamma coactivator 1-alpha (PGC-1α) promotes mitochondrial biogenesis, which may contribute to endothelial homeostasis (193). Curcumin has been reported to inhibit NF-κB signaling and cyclooxygenase (COX)/lipoxygenase (LOX) pathways, reduce TNF-α and IL-6 production, and attenuate inflammatory responses relevant to vascular injury (194–196). Emerging clinical observations have also been reported regarding effects on liver enzymes, insulin sensitivity, and early fibrosis markers; however, dedicated hepatic vascular endpoints were not assessed (197). Collectively, these agents may indirectly influence the vascular microenvironment by limiting oxidative and inflammatory stress; however, direct evidence from hepatic vascular endpoints remains limited.

### 4.3 Metabolic and anti-fibrotic agents with potential secondary effects on the vascular microenvironment

At advanced stages, progressive extracellular matrix accumulation and HSC contraction increase sinusoidal resistance and impair hepatic perfusion. Several agents that have advanced furthest in clinical development primarily target metabolic or fibrogenic pathways. While these interventions may indirectly benefit the hepatic vascular niche by reducing lipotoxicity, inflammation, or matrix stiffness, dedicated vascular or hemodynamic endpoints have generally not been measured in the pivotal trials. They are therefore discussed here as agents with potential secondary vascular effects rather than as direct vascular-targeted therapies.

Resmetirom, a selective thyroid hormone receptor-β agonist, reduces intrahepatic triglyceride accumulation and improves histological features of MASH with fibrosis (198–203). In March 2024, the U.S. Food and Drug Administration (FDA) granted accelerated approval to resmetirom (Rezdiffra) for the treatment of adults with noncirrhotic MASH with moderate to advanced liver fibrosis (stages F2–F3), to be used in conjunction with diet and exercise (198). The phase 3 MAESTRO-NASH trial demonstrated efficacy in achieving MASH resolution without worsening of fibrosis and in improving fibrosis outcomes (203). However, whether these metabolic and histological benefits translate into direct improvement of hepatic vascular function or microcirculatory parameters remains to be determined, as dedicated hepatic vascular and microcirculatory endpoints were not prespecified as primary efficacy endpoints in these pivotal clinical trials (203–205).

In addition to resmetirom, several other agents with potential secondary effects on the vascular microenvironment are under active investigation for MASH. Sodium-glucose cotransporter 2 (SGLT2) inhibitors, beyond their glucose-lowering effects, have demonstrated beneficial effects on endothelial function and vascular homeostasis, including improved endothelial-dependent vasodilation and reduced arterial stiffness (206). In MAFLD/MASH, accumulating evidence suggests that SGLT2 inhibitors may exert beneficial effects on hepatic metabolic abnormalities and fibrotic progression, although their effects on definitive histological endpoints remain under investigation (207). Aramchol, a partial inhibitor of stearoyl-CoA desaturase-1 (SCD1), has shown antifibrotic potential in phase 2 clinical studies and may indirectly influence the sinusoidal microenvironment by reducing lipotoxic stress and fibrogenic remodeling (208). Dedicated vascular or hemodynamic endpoints have not been systematically evaluated for these agents to date.

Metabolic surgeries, including Roux-en-Y gastric bypass and sleeve gastrectomy, have been associated with sustained weight loss, improved glycemic control and insulin sensitivity, and favorable hepatic outcomes, including resolution of steatohepatitis and reduced risk of advanced liver disease (209, 210). These systemic metabolic improvements may secondarily influence hepatic vascular homeostasis; however, direct evidence demonstrating restoration of sinusoidal function or reduction of intrahepatic vascular resistance after bariatric surgery remains limited.

Recent evidence supports the therapeutic potential of incretin-based strategies in MAFLD. The glucagon-like peptide-1 receptor agonist semaglutide has demonstrated efficacy in promoting MASH resolution and improving metabolic parameters in patients with MASH (211). Recent phase 3 ESSENCE trial data further demonstrated efficacy in patients with MASH and fibrosis (204). Similar benefits have also been observed with other GLP-1 receptor agonists, such as liraglutide (212). To date, these trials have not included direct assessments of endothelial function, sinusoidal perfusion, or portal pressure; any vascular benefit is therefore currently inferred rather than demonstrated.

### 4.4 Translational and clinical perspectives

Notably, virtually no completed phase 2 or phase 3 MASH trial has included endothelial function, nitric oxide bioavailability, sinusoidal perfusion, or portal pressure as a prespecified efficacy endpoint. This translational gap itself underscores the relevance of the vascular microenvironment framework proposed in this review.

Accordingly, future therapeutic development should incorporate vascular-specific endpoints to better evaluate treatment efficacy and to clarify whether metabolic improvements translate into restoration of hepatic vascular homeostasis. Potential endpoints include endothelial function, nitric oxide bioavailability, sinusoidal perfusion, portal hemodynamic parameters, and indicators of microvascular remodeling (26, 70, 213).

However, the clinical implementation of vascular assessments remains challenging. Although hepatic venous pressure gradient (HVPG) measurement provides a direct evaluation of intrahepatic hemodynamic alterations, its invasive nature and limited applicability in early-stage MAFLD—where clinically significant portal hypertension is often absent—restrict routine use (214). Therefore, future studies should prioritize the development and validation of minimally invasive approaches. These may include imaging-based assessments such as magnetic resonance imaging (MRI) evaluation of hepatic perfusion (215) and contrast-enhanced ultrasound (216), as well as emerging circulating biomarkers that reflect endothelial activation or microvascular injury, including extracellular vesicles and related markers (217).

Integrating these vascular assessments with metabolic, inflammatory, and fibrotic endpoints may enable a more comprehensive evaluation of therapeutic efficacy and support the development of precision strategies targeting hepatic microcirculatory dysfunction in MAFLD.

## 5 Discussion and future perspectives

Accumulating evidence has expanded the understanding of MAFLD from a primarily metabolic disorder toward a complex metabolic–vascular disease. The hepatic vascular microenvironment offers an integrative perspective that connects metabolic stress, immune activation, and tissue remodeling.

Current experimental studies suggest that endothelial dysfunction and sinusoidal abnormalities may occur during early stages of MAFLD and potentially contribute to subsequent inflammatory and fibrotic responses. Nevertheless, most mechanistic insights are derived from animal models and *in vitro* systems, whereas longitudinal human studies specifically evaluating hepatic vascular alterations remain scarce. In particular, the absence of validated vascular biomarkers and reliable approaches for monitoring sinusoidal dysfunction limits the clinical translation of the hepatic vascular microenvironment concept.

It is important to acknowledge the competing view that sinusoidal capillarization and endothelial dysfunction may largely represent secondary consequences of hepatocyte lipotoxicity, oxidative stress, and inflammatory activation rather than primary initiating events. In this alternative model, metabolic injury in hepatocytes drives the release of damage-associated signals and extracellular vesicles that subsequently impair LSEC phenotype and promote microvascular remodeling. Distinguishing a “vascular-first” from a “metabolic-first” model therefore remains challenging and will require prospective human cohorts that integrate vascular imaging, circulating vascular biomarkers, metabolic and inflammatory profiling, and longitudinal assessment of disease progression, together with complementary spatial omics and cell-type-specific experimental approaches. A balanced interpretation recognizes that vascular and metabolic processes are likely interdependent and may reinforce each other in a bidirectional manner, with the relative contribution of each varying by disease stage and individual susceptibility.

The stage-associated conceptual framework proposed here reflects stage-related biological patterns rather than a fixed temporal sequence. The relative contribution of vascular, metabolic, immune, and fibrogenic processes likely varies with disease stage, genetic background, and environmental factors.

From a translational perspective, a major challenge is determining whether vascular alterations can serve as clinically meaningful biomarkers or therapeutic endpoints. Although endothelial dysfunction and microcirculatory abnormalities have been extensively characterized experimentally, their relevance in human disease stratification and therapeutic evaluation requires further validation. Future clinical studies should prioritize feasible vascular assessments and integrate vascular-related parameters with conventional metabolic and fibrotic outcomes in clinical trials.

Overall, the hepatic vascular microenvironment provides an additional biological perspective for understanding MAFLD heterogeneity. Rather than replacing existing metabolic models, this framework highlights the importance of microcirculatory regulation and may facilitate the identification of new biomarkers and precision therapeutic strategies.
